# Paracentral Acute Middle Maculopathy

**DOI:** 10.4274/tjo.galenos.2020.92972

**Published:** 2020-06-27

**Authors:** Pelin Kıyat, Cumali Değirmenci, Serhad Nalçacı, Filiz Afrashi, Cezmi Akkın

**Affiliations:** 1 Ege University Faculty of Medicine, Department of Ophthalmology, İzmir, Turkey

**Keywords:** Paracentral acute middle maculopathy, acute macular neuroretinopathy, optical coherence tomography

## Abstract

Paracentral acute middle maculopathy (PAMM) is a variant of acute macular neuroretinopathy which is characterized by a hyperreflective band-like lesion in the inner nuclear layer and outer plexiform layer on spectral domain optical coherence tomography (SD-OCT). The etiology is believed to involve vasopressor exposure or systemic microvascular diseases that cause retinal ischemia. SD-OCT is the main imaging method in the diagnosis or evaluation of progression of PAMM, whereas multimodal imaging is useful to support the diagnosis. Herein, we present a case of PAMM in a healthy young woman using multimodal imaging methods.

## Introduction

Paracentral acute middle maculopathy (PAMM) was first described in 2013 by Sarraf et al.^1^ and was identified as a variant of acute macular neuroretinopathy. Although Bos and Deutman^2^ described acute macular neuroretinopathy as red, wedge-shaped paracentral retinal lesions, since the development of spectral domain optical coherence tomography (SD-OCT) it has been determined that some acute macular neuroretinopathy lesions affect the outer retinal layers while others affect the middle and inner retinal layers. PAMM, which was described as type 1 acute macular neuroretinopathy by Sarraf et al.^1^, is characterized by a band of hyperreflectivity in the inner nuclear and outer plexiform layers.^3^

Although its etiology has not been fully elucidated, causative factors may include the use of vasoconstrictors such as caffeine and epinephrine, oral contraceptive use, and microvascular diseases that affect the retina, such as diabetes, hypertension, and sickle cell anemia.^4,5^

## Case Report

A 21-year-old woman presented to the retina unit of the Ege University Faculty of Medicine Ophthalmology Department with complaints of blurry vision and the appearance of a black spot in her right eye for the past 4 days. Her medical history was unremarkable. On ophthalmological examination, her best corrected visual acuity (BCVA) was 20/20 in both eyes. Bilateral intraocular pressure and anterior segment examination results were normal. Fundus examination was unremarkable in the left eye but revealed a hypopigmented lesion involving the superotemporal fovea of the right eye.

SD-OCT imaging of the right eye demonstrated a hyperreflective band lesion at the level of the inner nuclear and outer plexiform layer corresponding to the hypopigmented lesion observed in the superotemporal fovea. The outer retinal layers and retinal pigment epithelium appeared completely normal. On 30-2 visual field test, a paracentral scotoma corresponding to the hypopigmented lesion on fundus examination was observed. Fundus autofluorescence imaging revealed hypoautofluorescence in the superotemporal foveal lesion and fundus fluorescein angiography revealed hypofluorescence in the same area. Substantial capillary dropout was detected in the deep capillary plexus on OCT angiography. No pathologies were detected on multifocal electroretinogram.

Hematology and genetics consultations, thrombophilia screening, hemogram and biochemical tests, carotid Doppler ultrasound, and cranio-orbital magnetic resonance imaging ordered as etiological studies revealed no abnormalities. The case was evaluated as suspected PAMM and the patient was followed without treatment.

**Figure 1:**
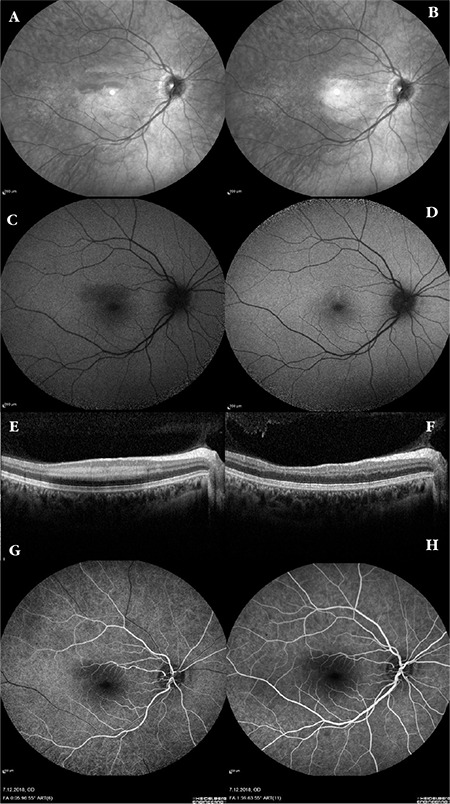
Fundus infrared, autofluorescence and SD-OCT and images at the time of diagnosis and at 4-month follow-up examination and FFA images at the time of diagnosis. In infrared images, hyporeflectance was detected in the superotemporal fovea lesion at the time of diagnosis (A) and was found to have regressed at 4-month follow-up (B). In fundus autofluorescence imaging, hypoautofluorescence was detected in the superotemporal fovea lesion at the time of diagnosis (C) and was found to have regressed at 4-month follow-up (D). SD-OCT performed in the right eye at time of diagnosis revealed a hyperreflective band lesion at the level of the inner nuclear and outer plexiform layer corresponding to the hypopigmented lesion observed in the superotemporal fovea (E). SD-OCT at 4-month follow-up demonstrated regression of the hyperreflective band including the inner nuclear and outer plexiform region in the superotemporal fovea, leaving retinal thinning and disorganization of the outer retinal layers in the lesion area (F). Early and late stage fundus fluorescein angiography images at the time of diagnosis (G-H). SD-OCT: Spectral domain optical coherence tomography

**Figure 2:**
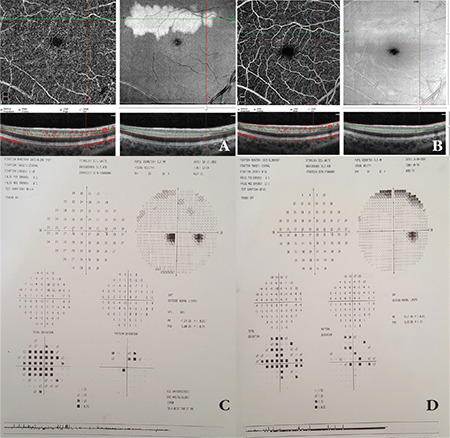
OCT angiography images and 30-2 visual field test at the time of diagnosis and at 4-month follow-up examination. OCT angiography at time of diagnosis demonstrated substantial capillary dropout in the deep capillary plexus (A). At 4-month follow-up, the capillary dropout was seen to have resolved, leaving disorganization in the deep capillary plexus (B). At diagnosis, 30-2 visual field test showed paracentral scotoma consistent with the hypopigmented lesion observed on fundus examination (C). On follow-up examination, the paracentral scotoma was found to have regressed (D). OCT: Optical coherence tomography

At 4-month follow-up, the patient stated that her vision had returned to normal and the black spot had completely disappeared. Her BCVA was 20/20 bilaterally, fundus examination demonstrated regression of the hypopigmented lesion, and SD-OCT revealed minimal retinal thinning and disorganization of the outer retinal layers in the area corresponding to the lesion in the superotemporal fovea. Regression of fundus hypoautofluorescence in the lesion area was observed (Figure 1). On OCT angiography, the capillary dropout in the deep capillary plexus in the lesion area was observed to have resolved, leaving disorganization of the deep capillary plexus. On 30-2 visual field test, the paracentral scotoma detected at diagnosis had regressed (Figure 2). No recurrence was detected in subsequent follow-up examinations.

## Discussion

Sarraf et al.^1^ described two variants of acute macular neuroretinopathy in 2013. Type 1 acute macular neuroretinopathy, also known as PAMM, is a form that involves the inner nuclear and outer plexiform layers on SD-OCT, whereas type 2 acute neuroretinopathy is the form that involves the outer retinal layers and retinal pigment epithelium.^6^

Both type 1 and type 2 acute macular neuroretinopathy lesions manifest with paracentral scotoma, and vasopressor exposure is implicated in their etiologies. In terms of clinical presentation, they both exhibit grayish hypopigmented intraretinal parafoveal lesions. The basic imaging method to distinguish between the two types is SD-OCT, and the source of ischemia is either the intermediate or deep capillary plexus in type 1 versus the deep capillary plexus in type 2. The parafoveal location of the lesions may be due to the density of the capillary plexus in this area.

In the literature, PAMM is usually reported in men with advanced vasculopathy, while type 2 acute macular neuroretinopathy cases consist of healthy young women.^7^ Unlike the cases reported in the literature, our patient was a healthy young woman with type 1 acute macular neuroretinopathy (PAMM).

SD-OCT is the main imaging method used to distinguish PAMM, which affects the inner nuclear and outer plexiform layers, from type 2 acute macular neuroretinopathy, which affects the outer retinal layers, ellipsoid zone, and retinal pigment epithelium.^8^ In some cases, ophthalmoscopic retinal examination findings can be completely normal and there may be no remarkable findings on fundus fluorescein angiography. In such cases, the importance of SD-OCT for diagnosis is clear. SD-OCT can be used to visualize the inner nuclear layer thinning that can occur in chronic cases, lesion progression, or resolution.^9^

Fundus fluorescein angiography imaging in our case revealed hypofluorescence in the parafoveal lesion area. Previously published cases of PAMM have generally been described as having normal fluorescence on fundus fluorescein angiography.^10,11^ In cases with branch retinal artery occlusion in the etiology of PAMM, filling defect has been detected on fundus fluorescein angiography.^3^ In our case, however, we observed no findings suggesting branch artery occlusion and filling defect was not detected. In their case report, Niyousha et al.^12^ observed hypofluorescence in the affected retinal area on fundus fluorescein angiography of the eye with PAMM, as in our case. We attributed the hypofluorescence observed on fundus fluorescein angiography imaging of our patient to a reduction in fluorescence due to retinal thickening.

Ischemia in the intermediate and deep capillary plexuses, which supply the intermediate retinal layers, plays an essential role in the pathogenesis of PAMM.^13^ Although we were unable to detect any etiological factor in our case, the presence of microvascular diseases in the etiology necessitates the evaluation of patients with PAMM lesions for microvascular diseases that may affect retina such as diabetes, hypertension, and sickle cell anemia. PAMM may be a warning sign of systemic microvascular disease.^14^

PAMM lesions have been associated with the use of sympathomimetics such as epinephrine, norepinephrine, ephedrine, and caffeine.^15^ Patients diagnosed as having PAMM must also be questioned about vasopressor exposure. Sympathomimetics may cause ischemia in the retinal intermediate and deep capillary plexuses due to their vasopressor effects and may be responsible for the hyperreflective lesion in the inner nuclear and outer plexiform layers observed on SD-OCT. The inner nuclear layer thinning that occurs in some patients with chronic PAMM supports the ischemia theory. In patients whose inner nuclear layer thins in the chronic period, paracentral scotoma may be permanent.^16^ In our case, the hyperreflective lesion observed on SD-OCT completely regressed and the scotoma resolved.

In conclusion, PAMM, which is defined as a variant of acute macular neuroretinopathy that affects the inner nuclear and outer plexiform layers, can be diagnosed more easily today owing to the development of SD-OCT and multimodal imaging methods. SD-OCT is a useful imaging method in the diagnosis, progression monitoring, and differential diagnosis of PAMM. The role of focal parafoveal ischemia in the retinal capillary plexus has been emphasized in the etiology of PAMM, and investigating vasopressor exposure and microvascular pathologies that may affect retina is recommended in patients diagnosed with PAMM. There is currently no treatment for PAMM lesions, but etiological research is important to identify systemic vascular risk factors.^17^
